# Nanobacteria: Facts or Fancies?

**DOI:** 10.1371/journal.ppat.0030055

**Published:** 2007-05-25

**Authors:** Pasquale Urbano, Francesco Urbano

**Affiliations:** The Scripps Research Institute, United States of America

## Introduction

An audacious theory proposes the existence of a novel form of life—the nanobacteria (NB)—that is quite different from the ones already known, but is capable of infecting and damaging other beings, thus qualifying them as new agents of emerging infectious diseases.

The theory is no less revolutionary than the famous germ theory of disease, which was put on solid ground by the efforts of Pasteur and Koch, or the one on *contagium vivum fluidum,* which heralded the birth of virology.

Other extraordinary findings have appeared in the recent scientific literature; some have opened new perspectives and have led to immense areas of new knowledge, while others, like cold fusion or the memory of water, have passed like meteors, and are remembered as minor happenings of little hindrance to the steady advancement of science.

What about NB? Are they going to be marked as milestones, or, as we fear, are they going to linger about for decades as UFOs have?

“Nanobacteria” is a neologism, introduced and patented by Dr. Olavi Kajander as the name for very small bacteria-like organisms. In 1998, a seminal paper in *PNAS* [1] by Kajander and Neva Ciftcioglu boldly announced the discovery of an unprecedented form of life having something in common with bacteria, but so much different from the ones already known that it deserved a new name. On account of their mineralizing properties, nanobacteria have also been called calcifying nano-particles [2].

Kajander and colleagues had worked for years on their invention, but their reports were turned down by the microbiological establishment because they went against accepted paradigms. The technical details of the methods originally used to define the key features of the purported novel organism have thus been presented orally, or published in obscure journals or proceedings, and their abstracts have often been taken uncritically at their face value. Galileo Galilei was vindicated by history, Kajander and Ciftcioglu so far only by the Web: in a Google search of July, 2003 they got 3,160 hits for “nanobacteria”; with the same keyword, we now (as of January 8, 2007) get 90,500 hits with Google, and 198,000 with Yahoo.

Another obstacle to the equitable assessment of the evidence is the fact that its main contributors have—legitimately—started a diagnostics and pharmaceutical business enterprise, and their papers do not always state their business connections.

## Distribution and Disease Association

NB have been reported to be present in animal [3,4] and human blood [5], in bile [6], in tissue culture cell lines [7], in wastewater [8], in Australian sandstones, in the stratosphere [9], and in meteorites [10]. Most of the reports are based on the visualization of nanobacteria by scanning electron microscopy, but some are supported by their propagation in cell-free media, according to the inventors.

Whereas some of the findings have given rise to highly speculative lines of thought, with hypotheses about their extraterrestrial origin or their primordial role in the development of life on Earth, we are more interested in the nature of NB and in their purported association with pathological conditions.

In line with the experimental demonstration that NB are efficient nuclei of mineralization that start the formation of apatite from soluble calcium and phosphorus compounds at physiologic concentrations and conditions, many authors have followed Kajander's lead [1,11,12] and reported NB in association with a variety of pathological calcifications, notably nephrolithiasis [13,14], cholecystolithiasis [15], vascular plaques [16,17], valvular calcification [18], psammoma bodies in ovarian cancers [19], mammary cancer [20] and breast implant contracture [21], osteoarthritis [22], chronic prostatitis and prostate stones [23], and periodontal disease [24]. NB have also been implicated in intervertebral disk degeneration [25], polycystic kidney disease [26,27], reduced bone density in HIV [28], and peripheral neuropathy [29].

It is a fact that the process of pathological calcifications is largely unknown, and the idea that it might be due to some novel form of life is appealing but provocative. Most of the cited reports assume that NB do exist and that they are living organisms with the peculiar properties described by Kajander and collaborators; few of them, notably the papers by Cisar et al. [30] and Drancourt et al. [31], challenge the assumption; only the paper by Miller et al. [17] brings new and independent experimental data to support it.

## Ontology of NB

According to their discoverers [7], NB are very minute bodies ranging in size from 20 to 500 nm, the smaller ones filterable through membranes with 100-nm pores, and are observable by scanning electron microscopy or transmission electron microscopy, where they appear as spheres or rods. Many others have observed such bodies in diverse substrates and have called them nanoforms, nannobacteria, nanoparticles, nano-organisms, nanobes, living nanovesicles, microfossils, etc. The hallmark of NB is their more or less thick coat of apatite, which is formed from soluble calcium and phosphorus compounds in their environment or medium.

Ciftcioglu and Kajander came across NB as cytopathic contaminants of cell cultures; their early findings are recapitulated in a 1998 paper [7], which emphasizes the fact that traditional microbiological methods fail to reveal NB, and that specific methods had to be invented for their detection and culture; they had already patented such methods in 1992. In short, they developed a new culture, DNA staining, and immunoassay methods, by which they convinced themselves, and tried to convince others, that they had discovered a new living organism, or at least “the smallest culturable autonomously replicating agent on Earth” [32]. This very claim was theoretically audacious [33,34], and it instigated the reaction of traditional biologists, who reached a consensus about the admissible lower size limit of a microorganism [35]; the idea that the machinery necessary for sustaining life as we presently know it could fit in a 20-nm sphere was flatly rejected.

Another key trait of Kajander's NB is their apparent culturability in cell-free media, typically the ones normally adopted for cell cultures, like Dulbecco minimal essential medium or RPMI-1640, with or without fetal calf serum or other supplement. This kind of propagation has succeeded in the hands of many independent researchers, notably of Cisar et al. [29], who, however, give an alternative interpretation of NB-induced mineralization, and find no evidence for the living nature of NB. Experienced microbiologists who had failed to substantiate the positive findings [31] received a piquant rebuttal [36].

The filterability of NB is another stronghold of the NB addicts; of course, there is no question about nano-sized particles being able to go through 0.1-μ pores. The point is their being able to replicate in the sense which is normally given to the word. The prion scrapie protein is filterable and it will propagate in the presence of the normal prion protein, but not by binary scission, as bacteria do. At any rate, the filterability of microorganisms is a long-lasting and unsettled argument [37].

NB are said to be immunogenic, and kits for the detection of their antigen or antibodies have been developed by NanoBac Oy, Kuopio, Finland; the specificity of the proprietary immunological reagents is controversial, as is the evidence based on their use.

New experimental data by Miller et al. [17], working with NB propagated from vascular calcifications, have been construed to demonstrate the organismic nature of NB on the basis that they contain DNA, synthesize proteins and at least one NB antigen, and incorporate uridine, albeit at very low levels, barely above the negative controls.

Almost nothing is known about the metabolism of NB; they are said to double in one to five days, mostly on the basis of rising optical density or of increasing mineral mass. Nucleic acid presence and metabolism is controversial, as standard methods are said to be inadequate to their study. The sequence of a putative 16S rDNA PCR amplicon was hastily proposed as the basis for establishing a new genus and species, *Nanobacterium sanguineum,* which was placed in the α-2 subgroup of Proteobacteria, which also includes *Brucella* and *Bartonella* species. The European Molecular Biology Laboratory (EMBL) X98418 and X98419 files in the EMBL database cannot be opened at present, while a search in the Deutsche Sammlung Von Mikroorganismen Und Zellkulturen (DSMZ) catalogue for the material deposited with number 5819-5821 yields no answer.

After Cisar et al. [30] suggested that the published sequence came from a contaminant, it became clear that there is no sound basis for the existence of *Nanobacterium sanguineum,* and the name has no standing in the nomenclature. Kajander himself recently admitted that the bacterial status of NB is still lacking satisfactory evidence, and he concedes that the term “calcifying nanoparticle” best describes the agent [38]; unfortunately, he also still uses the untenable word “nanobacteria”, which landed him in a microbial minefield, as an editorial had rightly foreseen [39]. In his retreat he establishes a second line of defence, insisting that calcifying nanoparticles are infectious agents, not Eubacteria or Archea, but entities of their own, perhaps of primordial origin. In this he follows the wild hypotheses of Sommers, a prolific writer whose papers deserve a review of their own [28,29].

We try to keep an open mind and we accept the idea that new evidence may extend the limits of our knowledge, and even overturn long-accepted paradigms; however, we believe that in so doing it is not acceptable to misuse words and concepts that have their foundation in the current paradigm. To be clear, it is a fact that in Kajander's *PNAS* paper [1], Figure 1E depicts an unknown bacterium, and that other, seemingly different, bacteria are depicted in various papers by the Nanobac group; nothing proves that they are one and the same entity as the much smaller pleomorphic nanoparticles. It is also a fact that nothing has been done to identify or to characterize it, or them; nowhere have we found that NB have been “isolated”, and we know that in the mid-1800s, the revolutionary germ theory of disease would not have been accepted without the cumbersome methods set up by Pasteur for obtaining pure cultures, and it would not have gained impetus without the isolating cultures on the solid media of Koch.

Also untenable is the idea that any trait observed on NB, whatever their source, may be attributable to a single entity, and that this piecemeal evidence, every piece of which is debatable, can ever lead to sound knowledge. For instance, how does one reconcile the report that the cell wall of NB is twice as thick as that of Staphylococcus epidermidis [40], with the often-cited Gram negativity of NB? And what is the basis for their asserted motility? The catalase negativity—in fact, any enzyme negativity whatsoever—adds nothing to what we know about NB.

## Risk and Disease Association

The inventors of NB strongly promote the idea that they are infectious pathogens and that they represent a global health risk. However, there is a problem with the semantics, the concept of infection being stretched beyond its traditional meaning of parasitic symbiotic relationship. Still, pragmatism forces us to consider that calcifying particles could in fact be involved in pathological processes, serving as crystallization nuclei. In view of the quasi-ubiquity of NB, proof of their etiologic or pathogenic role is going to be a formidable task, to be repeated for each and every disease; the associations already reported are at best to be considered working hypotheses, to be confirmed by independent research with non-proprietary methods and reagents.

Shortcuts are risky; in the era of evidence-based medicine the time-honoured *ex iuvantibus* criterion must be exercised with rigid and formally explicit requisites. The two papers claiming therapeutic success with a complex anti-NB regimen that includes comET, a patent “nutraceutical”, are not randomized, controlled, or properly blinded. In particular, the paper by Maniscalco and Taylor [41] states that 57% of the treated patients had decreased coronary artery calcification scores; these patients were labelled “responders”, and among “responders”, the scores where significantly lower! Nothing is said about the rest of the study group, and one is left with the certainty that they had (by definition) stable or increased scores, perhaps significantly so; such a grossly misleading presentation of the data verges on quackery.

The paper by Shoskes et al. [23] is a small open label pilot study; the authors refer to “the complete resolution of concomitant CPPS [chronic prostatitis/pelvic pain syndrome] symptoms” anecdotally reported by Maniscalco, before the publication of the cited paper, but the results are presented more prudently, and the relationship with Nanobac Life Science is correctly declared.

The reported wide distribution of NB, their filterability, and their asserted pathogenicity are strong selling points for the NB business. The pharmaceutical industry is urged to look for NB, and to invest in their elimination; vaccines, blood, and blood derivates should be guaranteed NB-free. Space flights should take into account that NB grow faster at microgravity [42], and the US National Aeronautics and Space Administration already worries about it.

The reported consistent prevalence of NB in the blood of healthy people is presented as a risk factor for a number of pathologies. The lay press comes out with best sellers like *The Calcium Bomb,* and the Web acts as a bandwagon.

In short, we are experiencing an aggressive risk-mongering and disease-mongering campaign, and journal referees have been, are, and will be hard pressed with papers that mix NB facts with NB fancies; the papers they reject are going to swell the grey literature, and blogs will be filled with pieces condemning the obscurantism of the non-believers. 

## Abbreviation

NB: nanobacteria
